# Emotion recognition bias depends on stimulus morphing strategy

**DOI:** 10.3758/s13414-022-02532-0

**Published:** 2022-07-05

**Authors:** Anastasia Vikhanova, Isabelle Mareschal, Marc Tibber

**Affiliations:** 1 Department of Psychology, School of Biological and Chemical Sciences, Queen Mary University of London, London, E1 4NS UK; 2 Research Department of Clinical, Educational and Health Psychology, UCL, London, UK

**Keywords:** Emotion recognition, Morph, Depression, Anxiety, Cognitive bias

## Abstract

Emotion recognition is vital for social interactions, and atypical (or biased) emotion recognition has been linked to mental health disorders including depression and anxiety. However, biases in emotion recognition vary across studies, and it is unclear whether this reflects genuine group differences in psychological processes underlying emotion recognition or differences in methodologies. One common method to measure biases in emotion recognition involves morphing a face between two emotional expressions in different ratios and asking participants to categorise the faces as belonging to one of the two emotion categories (‘direct-morphing’ method). However, this method creates morphed faces that are not ecologically valid. Alternatively, faces may be morphed through a neutral expression (‘morphing-through-neutral’ method), which is more ecologically valid since emotional expressions usually start from a neutral face. To compare these two approaches, we measured emotion recognition biases using two morphing techniques in 136 participants who also completed measures of anxiety (GAD-7) and depression (PHQ-9). Biases obtained using the two methods differed significantly: In the direct-morphing method, participants perceived the central 50% happy/50% angry face as slightly happy, whereas in the morphing-through-neutral method the neutral face was seen as angry. There were no associations between biases and depression or anxiety scores for either morphing method. This study is the first to directly compare emotion recognition biases obtained using two different morphing methods and is a first step towards reconciling discrepancies in the literature.

**Supplementary Information:**

The online version contains supplementary material available at 10.3758/s13414-022-02532-0.

## Introduction

Emotion recognition forms the foundation of healthy social communication (Blair, 2005). Facial emotions convey critical information about state of mind (Baron-Cohen, 1997) and an inability to interpret emotional expressions is associated with a number of clinical disorders (e.g., Capitão et al., 2014). Emotion recognition biases – the systematic tendencies people have to perceive ambiguous expressions negatively or positively – are often associated with mental health disorders such as depression and anxiety, where they have been suggested to play a key role in their formation and maintenance. For example, systematically misjudging facial expressions as negative can lead to an avoidance of social communication and subsequent social exclusion, which in turn impacts mental health (Harmer et al., 2009).

However, findings for both clinical and non-clinical populations are often mixed in terms of the sign/direction of emotion recognition biases (Bell et al., 2011; Münkler et al., 2015). For example, Leppänen et al. (2004) found that healthy participants did not exhibit a bias when presented with neutral faces. In contrast, Surguladze et al. (2004) found that healthy participants perceived neutral faces as happy, while depressed participants perceived them as neutral. It is unclear whether these mixed results reflect genuine individual differences in perception of emotions, or whether they reflect methodological differences.

**Fig. 1:**
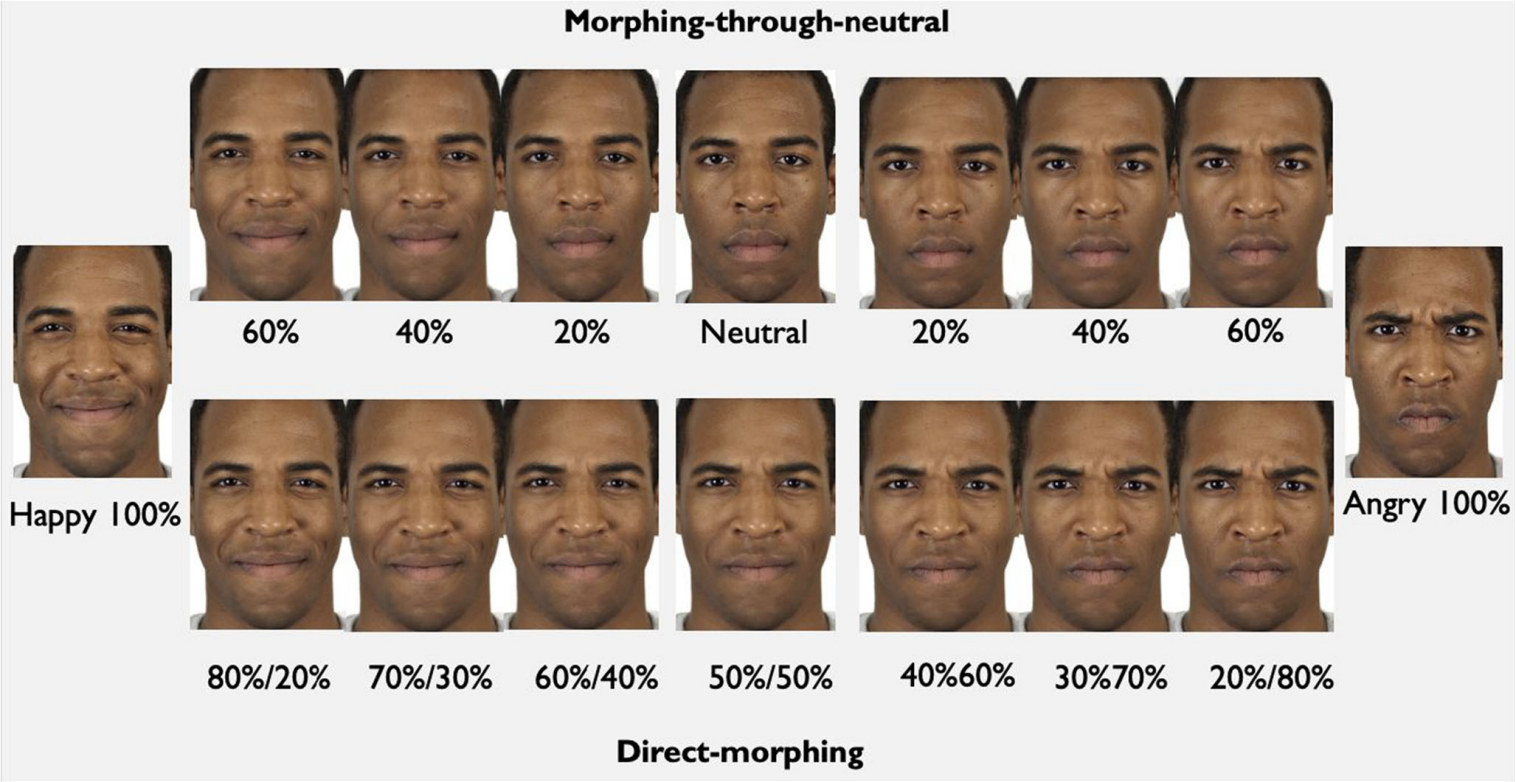
Black male actor’s morphed facial expression between 100% happy and 100% angry using two types of morphing

One common method for measuring biases in emotion recognition involves morphing same-identity images between two extreme emotions. This method creates faces of varying (emotional) ambiguity, with those around the equal ratio morph level appearing the most ambiguous (Liu et al., 2012; Maoz et al., 2016; Qiu et al., 2018). If participants systematically categorise these ambiguous faces as appearing as one of the emotion categories, they are said to be biased towards that category. However, this method creates unrealistic faces that are composites of expressions that may be discordant, and never co-occur (Paiva-Silva et al., 2016). The central morphed faces (Fig. 1, lower panel) often have odd expressions, and the measured bias might reflect individual and cultural differences with respect to which facial areas/features participants focus on, since different parts of the faces are most important for different types of expressions (Elfenbein & Ambady, 2002; Schurgin et al., 2014).

Alternatively, it is possible to include the neutral face as a central point for morphing towards either extreme emotion (Michalek et al., 2021). Introducing a neutral face in the morphing procedure means that morphed faces will only contain elements of a single expression combined with the neutral face (Fig. 1, upper panel). This is an alteration of dynamic morphing methods, where the faces presented change from neutral to emotional and the participant must categorise the emotion as soon as they identify it (Kessels et al., 2014; Paiva-Silva et al., 2016). The theoretical use of a neutral face as a natural mid-point between expressions is supported by after-effect studies. Russell and Fehr (1987) showed that a neutral face would be seen as sad when presented next to a happy face, whereas it would be perceived as happy when presented next to a sad face. Rutherford et al. (2008) extended these findings to demonstrate an emotion ‘after-effect’, whereby a neutral face that is presented immediately after a happy face appears sad (and vice versa). Thus, the fact that adapting to sad faces makes the neutral appear happy (and vice versa) suggests that positive and negative emotions might be encoded on a continuum that goes through the neutral face, strengthening support for a morphing method that involves neutral faces.

Although the pros and cons of using different types of stimuli (including morphed and unaltered) in emotion recognition tasks have been discussed (Barrett et al., 2019; Paiva-Silva et al., 2016), no one to our knowledge has directly compared the two morphing methods discussed above. By comparing morphing approaches within a single sample of healthy participants we can gauge the extent to which variations in methodology potentially contribute to inconsistencies in the literature. Therefore, the first aim of the current study was to compare emotion recognition biases using morphed emotional expressions between anger and happiness (hereafter referred to as direct-morphing) and between anger and happiness going through a neutral expression (hereafter referred to as morphing-through-neutral). We focused on happy and angry faces as this was part of a larger study on threat perception, and these are commonly used to represent non-threatening and threatening stimuli, respectively. Due to previous inconsistencies in the literature, we tested the null hypothesis (H1) that there would be no significant difference in emotion recognition biases using the two morphing methods.

Second, we explored associations between biases and common mental health symptoms (anxiety and depression) in order to test whether such widely documented associations depend on the morphing method used. More specifically, if biases using these two types of morphing yield the same results, that would suggest that differences in stimulus methodologies might not be responsible for the previously reported mixed results. Thus, we tested the null hypothesis that (H2) emotion recognition biases using both methods would be significantly correlated with depression and anxiety scores. However, given that healthy participants have been found to exhibit positive emotion recognition biases (Surguladze et al., 2004), negative biases (Attwood et al., 2017) and no biases at all (Leppänen et al., 2004), we refrained from proposing a directional hypothesis for either mental health measure.

## Method

The study was approved by the ethics board of Queen Mary University of London (QMERC2019/70). Participants were recruited through an online recruitment platform Prolific (www.prolific.co), gave written informed consent to take part, and were paid £2.35 for participation (calculated at a £7 per hour rate).

### Stimuli and morphing procedure

Neutral, happy and angry expressions of 20 actors were selected from the Chicago Face Database (CFD; Ma et al., 2015) to create four test identity groups (five actors per group): Black female, Black male, White female and White male. The selected actors’ age range was 19–27 years to approximate that of our participants. Faces did not differ on attractiveness, trustworthiness or dominance, and all actors’ neutral faces were correctly classified as neutral as reported by the CFD authors (Ma et al., 2015). We selected faces of two ethnicities because this was part of a larger study (not presented here) to test differences in emotion recognition bias when participants judge faces of their own versus another ethnic group. However, we were unable to include South Asian faces despite having a South Asian participant group, as there are currently no databases available that include a full range of validated emotional expressions for South Asian actors.

We cropped all faces to 736 × 1,080 pixels using Adobe Photoshop and then morphed them with Abrosoft FantaMorph Deluxe 5 along two continua: angry-happy (direct-morphing) and angry-neutral-happy (morphing-through-neutral). For the former, each of the faces was morphed in 10% steps between happy and angry expressions, resulting in a total of 11 faces (with 50% happy and 50% angry in the middle). For the latter, each of the faces was morphed in 20% steps between happy-neutral and angry-neutral expressions, resulting in a total of 11 faces (with the neutral face in the middle).

Since we were mainly interested in participants’ perception of the ambiguous faces, we removed some of the higher intensity morphs (80% happy/20% neutral, 80% angry/20% neutral for morphing-through-neutral, and 90% happy/10% angry and 90% angry/10% happy for direct-morphing) to reduce the number of trials. An example of the same actor’s face morphed using two methods can be seen in Fig. 1. For data analysis purposes, we label the happy faces with positive values (e.g., 100% happy = +100) and the angry faces as negative (100% angry = -100). The full list of selected faces is available in the Online Supplementary Materials (OSM, Table S1).

### Self-report measures

Participants provided basic demographic information including age, gender and ethnicity. Following this, and as part of a larger study, participants completed three questionnaires in randomized order: Perceived Ethnic Discrimination Questionnaire (Contrada et al., 2001; not discussed here), anxiety (GAD-7) and depression (PHQ-9).

### Anxiety (GAD-7) and depression (PHQ-9) questionnaires

To assess anxiety and depression, we used the seven-item Generalized Anxiety Disorder Scale (GAD-7; Spitzer et al., 2006) and the nine-item Patient Health Questionnaire (PHQ-9; Kroenke et al., 2001), respectively. Both measures are self-report questionnaires designed to assess mental health status during the previous 2 weeks. Items quantified how often a person was bothered by problems such as feeling nervous, worrying, troubles relaxing as anxiety measures; and feeling down, troubles sleeping, tiredness as depression measures on a four-point Likert scale from 0 (‘not at all’) to 3 (‘nearly every day’). Both questionnaires have equivalent cut off scores with 0–5 representing mild, 6–10 representing moderate, 11–15 indicating moderately severe, and scores above 15 signifying severe anxiety/depression. These scales have been widely used for clinical and non-clinical populations, and have high validity, reliability, and diagnostic sensitivity and specificity for clinical and general populations (Kroenke et al., 2001; Löwe et al., 2008; Martin et al., 2006; Spitzer et al., 2006). In the current study, Cronbach’s alpha for GAD-7 was 0.87, and 0.90 for PHQ-9 indicating high internal consistency.

### Eligibility criteria and procedure

The experiment (both questionnaires and the task) was designed and presented using Gorilla Experiment Builder (www.gorilla.sc; Anwyl-Irvine et al., 2020). Participants were recruited through the online platform Prolific (www.prolific.co) and took part on their own personal computers and laptops, and were not able to access the experiment on any other devices. Inclusion criteria included being a student currently resident in the UK aged 18–33 years. In addition, as part of the broader project, participants were specifically recruited from the following ethnic groups: (i) Black (Black Caribbean, Black African, mixed, other), (ii) South Asian (Asian/Asian British Indian, Pakistani and Bangladeshi, other), and (iii) White participants born in any EU country (excluding Ireland and Malta, where English is one of the official languages).

Participants first provided consent, then completed demographic questions and the three questionnaires (PEDQ, GAD-7, PHQ-9). To ensure participants paid attention, several attention checks were included in the questionnaires, e.g., “if you are reading this question, please select ‘not at all’”. Participants were excluded if they failed to respond correctly to any attention check.

Following completion of the questionnaires, participants completed Gorilla’s in-built calibration procedure such that all images were presented at a width of 7 cm on their displays, whilst maintaining the aspect ratio. After calibration participants performed the emotion-recognition task. Participants were instructed that they would see faces of different men and woman and their task was to indicate whether the face looked happy or angry using key-presses of ‘A’ key for angry, and ‘H’ for happy. The experiment was self-paced, and the next trial started when the participant provided a response to the previous. However, participants were asked not to dwell on each image and to make a guess if they were unsure of the emotion. Participants completed five practice trials prior to starting the task.

**Fig. 2:**
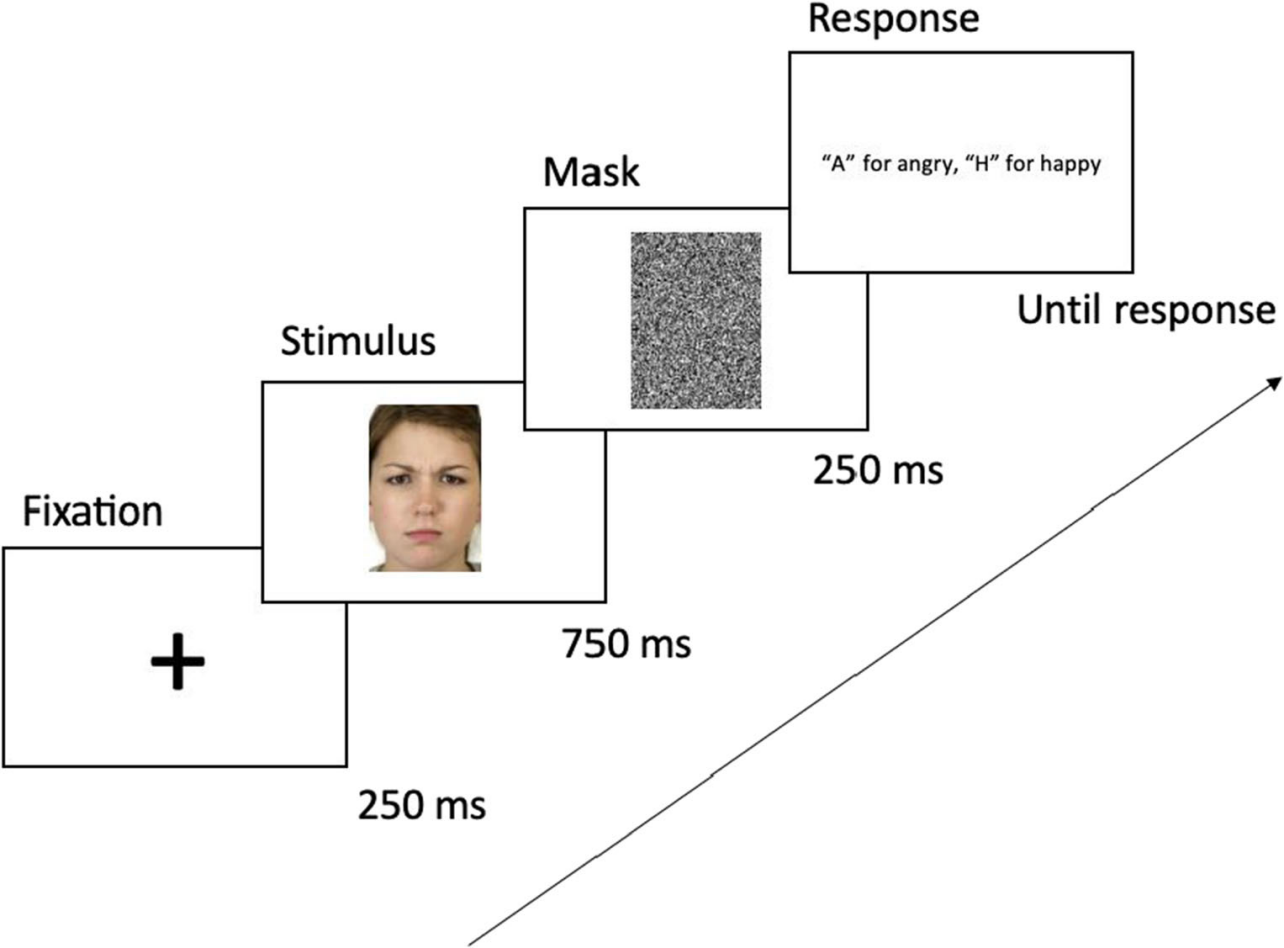
Timeline of a single trial with a White female face at 60% angry/40% neutral morph level from the morphing-through-neutral method

Each trial began with a 250-ms fixation cross, followed by an image of a test face for 750 ms. A white noise mask the same size as the face immediately followed for 250 ms after the test face was extinguished to avoid after images and carry-on effects (visual perseverance), followed by the response screen. Participants were only able to respond during the response screen that provided instructions about the key presses. The response screen disappeared after participant response (Fig. 2). Stimuli were presented in a randomised order. For both morph methods, the 100% happy and 100% angry images were identical, resulting in 14 morphed images per actor identity plus their two 100% (unmorphed) expressions. There were five actors per test identity, and four test identities, resulting in a total of 320 images run in two blocks of 160 trials. All faces from both morphing continua were randomly interleaved and only shown once. At the end of the experiment, participants were debriefed and received their Prolific completion code for payment.

### Task data analysis

We calculated the percentage of happy responses for each participant, separately for the four test identities (with five repeats/morph level representing the five actors). The data were fit with a cumulative Gaussian psychometric function in MATLAB (2018) and the point of subjective equality (PSE) was extracted corresponding to the morph level leading to a 50% ‘happy’ response (e.g., the morph level for which the participant was equally likely to perceive the face as happy or angry). We used the PSE as an index of participants’ bias. A positive PSE indicates that a slightly happy morph face is equally likely to be labelled happy or angry, and hence that the central morph face is more likely to look angry. Therefore, a positive PSE represents a bias towards an angry expression, and a negative PSE represents a bias towards a happy expression. In the direct-morphing method, an unbiased PSE would have a value of 50 (rather than 0), so we subtracted 50 from PSE scores to normalize the data across the two morph conditions. To obtain a gender- and race-independent measure of emotion recognition bias per morphing procedure, the data for the four test identities (i.e., Black female, Black male, White female, White male) were averaged.

Data were excluded if the bias (PSE) was above 80 or below -80 as this would suggest that a participant was unable to differentiate or recognise a full intensity emotional expression, as well as data where the threshold (slope) was larger than 200 (as the intensity of emotion ranged from -100 to +100 and therefore could not be larger than 200). Since for each morphing method there were four test identities, if the participant failed on the above criteria on more than one identity, their entire data set was excluded. This resulted in the removal of 0.04% of data in total.

Since participants were not given instructions to respond as quickly as possible, response data are not presented. However, figures depicting response times across different morph levels for both morphing methods can be found in the OSM (Figs. S1 and S2). Mean response times were calculated after removing outliers falling above three standard deviations (*M* = 581.63 ms, *SD* = 1,948.30 ms). This resulted in the removal of 0.38% of the data in total. The response times for central faces or any other morphing levels did not differ between morphing-through-neutral and direct-morphing (all *ps* > .05).

## Results

### Participants

An a priori power analysis was conducted using G*Power3 (Faul et al., 2007) to calculate the minimum sample size for a two-tailed difference between two dependent means (within-participant design) of emotion recognition biases using two morphing types, assuming a small-to-medium effect size (*d* = 0.30) at an alpha of .05 and power of 0.90 (based on previous literature). Results indicated a minimum sample of 119 participants, but since we aimed to recruit three groups as part of a larger study, we collected the data from at least 150 participants (50 per group).

One hundred and fifty-two participants (52% females, 47% males, 1% other) aged 18–33 years (*M* = 21.75, *SD* = 3.42) took part in the study. Sixteen participants were excluded due to technical error (*n* = 1), failed attention checks (*n* = 10) or poor performance on emotion recognition task (*n* = 5); thus, the final sample included 136 participants (35% South Asian, 33% White, 32% Black). According to self-report measures, participants reported mild-to-moderate levels of anxiety (GAD-7; *M* = 8.25, *SD* = 5.03, range 0–21) and moderate depression (PHQ-9; *M* = 10.53, *SD* = 6.73, range 0–24).

### A comparison of emotion recognition bias using two morph methods

**Fig. 3:**
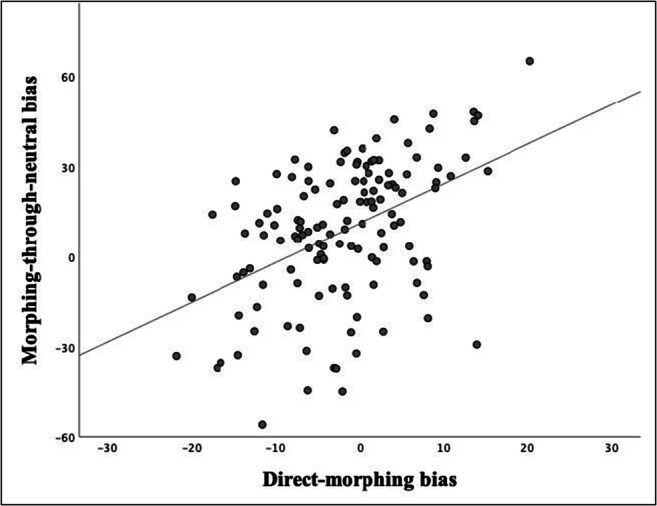
Correlation between direct-morphing and morphing-through-neutral emotion recognition biases. Each point represents one participant

To test our first hypothesis, we ran a repeated-measures t-test and found a significant difference between the direct-morphing and morphing-through-neutral methods, *t*(132) = 5.87, *p* < .001, *d* = 0.61. Further, Pearson’s bivariate correlation analysis with bias-corrected and accelerated (BCa) bootstrap interval procedure with 1,000 repeats revealed a significant positive relationship between biases obtained using the two types of morphs, *r =* 0.45, *p* < .001, 95% CI [0.28, 0.60] (Fig. 3).

We ran Bonferroni-corrected one-sample t-tests to investigate the direction of the bias for each method and whether it was significantly different from the central morph (e.g., an unbiased PSE). We found a significantly negative PSE (*M* = -2.06, *SD* = 7.98) for the direct-morphing method (bias towards happy faces), *t*(134) = 3.00, *p* = .003, *d* = 0.26, but a significantly positive PSE (*M* = 8.03, *SD* = 23.99) for the morphing-through-neutral method (bias towards angry faces), *t*(133) = 3.88, *p* < .001, *d* = 0.33.

### The relationship between emotion recognition bias and depression/anxiety

Pearson’s bivariate correlation analyses with BCa bootstrap interval procedure with 1,000 repeats were conducted to investigate the relationship between emotion recognition biases obtained using the two morph types and measures of anxiety and depression. Bonferroni corrections were made for two comparisons (correct alpha = .025) reflecting emotion recognition bias for two types of morphing within each questionnaire measure. Neither biases measured using direct-morphing nor biases measured using morphing-through-neutral were significantly associated with depression or with anxiety scores (all *ps* > .600) (see OSM Table S2).

## Discussion

The aim of the current study was to examine whether different stimulus generation methods (direct-morphing and morphing-through-neutral) would lead to systematic differences in estimated emotion recognition biases. Contrary to our null hypothesis (H1), we found a significant difference between the two methods: Participants exhibited a bias to perceive neutral faces as angry for the morphing-through-neutral stimuli, but exhibited a small, but statistically significant, bias to perceive the 50%/50% happy-angry morph as happy for the direct-morphing stimuli. Despite this, biases obtained using the two morph types were significantly (positively) correlated. A secondary aim was to investigate whether these biases were associated with measures of anxiety and depression. Contrary to H2, we found no significant relationship between either psychopathology measure and the biases using the two morph methods.

When considering the results of the two morphing methods separately, it is perhaps unsurprising that morphing through neutral yielded a mean negative bias. This is consistent with earlier reports that healthy participants often perceive neutral faces as negative (Lee et al., 2008) or angry (Carré et al., 2010). Surprisingly, we found a bias in the opposite direction when using stimuli from the direct-morph method, that is the 50/50% angry/happy faces were typically perceived as happy. It is known that happy faces usually ‘pop out’ when embedded amongst neutral distractors, whereas angry faces do not pop out (Becker et al., 2011; Juth et al., 2005). It is possible, therefore, that even a slight presence of a smile on an angry-happy morphed face might lead to the ambiguous face being identified as happy by attracting the participants attention to the smile.

It is difficult to determine if the differences in findings we report with respect to morphing methods underlie existing discrepancies in the literature since to our knowledge no study has compared the two morphing methods in healthy populations. For example, few studies used the morphing-through-neutral method to obtain a measure of bias. One of the largest normative studies on healthy participants (Kessels et al., 2014) investigated emotion recognition in dynamically morphed faces between neutral and emotional expressions of varying intensity, but that does not allow for an easy comparison with our task since it does not provide an estimate of bias. Specifically, their task required the participant to indicate what emotion they perceived when the facial expression changed from neutral to an emotion. This method therefore provides an estimate of the minimum facial configuration change required to recognise an emotion, but this is not a measure of bias.

There are substantially more studies using direct-morphing methods, although often with mixed results. Liu et al. (2012) using a similar direct-morphing procedure and study design found that on angry-happy continua depressed patients exhibited a bias towards angry expressions, which was larger than for healthy controls. However, Yoon et al. (2014) revealed that both high and low social anxiety groups had a higher rate of mislabelling faces as happy, thus exhibiting a bias in the opposite direction to Liu et al. (2012). Reviewing the literature on direct morphing methods, it appears that on balance most studies report a bias towards angry expressions in both clinical and non-clinical populations, although there remain discrepancies in findings.

Despite the difference in the direction of the emotion recognition biases yielded by the two morphing methods, we found a positive relationship between them, such that a larger anger bias in the morphing-through-neutral method was associated with a smaller happiness bias in direct-morphing method. This is consistent with common or at least overlapping mechanisms underlying biases as measured using the two morphing stimuli sets. However, our results suggest that findings in the literature should be interpreted with caution as the sign of estimated biases is dependent on the stimulus generation method. New effective methods of measuring emotion recognition are emerging, such as EmoGen (Roubtsova et al., 2021), whereby participants are given an opportunity to generate subjective emotional expressions on a 3D avatar using genetic algorithms, which provide insight into people’s internal representations of emotions (Carlisi et al., 2021).

It is not quite clear why the biases (using either stimulus generation method) did not correlate with measures of anxiety, as previously reported. Although it is uncommon to have a bias towards anger in depression (as compared to a bias towards sadness, e.g., Bourke et al., 2010), we expected to find a relationship between a bias towards anger and anxiety. For example, Attwood et al. (2017) used same-identity faces morphed between happy and angry expressions and experimentally induced anxiety in half of their healthy participants. They found that all participants exhibited a bias towards perceiving ambiguous expressions as angry, though this was elevated in the anxiety-induced condition. In our study, data collection was conducted during the COVID-19 pandemic and a national UK lockdown, which has seen an increase in mental health difficulties across all healthy populations (Pfefferbaum & North, 2020). Our participants presented moderate levels of depression and mild-to-moderate levels of anxiety, which are higher than previous averages in the UK student population (Thorley, 2017). Therefore, and in line with previous studies, we expected heightened emotion recognition biases towards anger. However, this is not what we found. One possible explanation is that depression and anxiety scores were generally inflated and hence showed ceiling effects at the time of data collection, leading to a reduced likelihood of finding an association between variables.

There are a number of limitations to this study. First, due to time constraints (or the expected experiment completion time), we were unable to include sad morphs, which would have been better suited to probe an emotion bias associated with depression. Second, it could have been useful to include measures of other mental health symptoms. For example, many of the previous studies on emotion recognition bias in anxiety have used the Social Interaction Anxiety Scale (SIAS; Heimberg et al., 1992), which provides a measure of *social* anxiety. Socially anxious patients are particularly attuned to social threat, for example threatening facial expressions (Mogg & Bradley, 2002), and exhibit a larger anger bias compared to healthy participants (Maoz et al., 2016). We selected generalised clinical measures (GAD-7) to decrease testing time, but this does not include measures of social anxiety, which may be critical. Finally, due to constraints of COVID-19 pandemic, the testing took place online, which would have resulted in noisier data than in laboratory-based studies.

## Conclusion

This study is the first to investigate emotion recognition biases in a healthy population, directly comparing two methods of stimulus generation. We found that the two methods led to different group mean biases, whereby neutral faces were (typically) perceived as angry, while 50%/50% happy-angry morphs were perceived as slightly happy. Importantly, we found that biases using the two types of stimuli were significantly positively correlated, suggesting that emotion recognition biases can be reliably measured using different types of stimuli, but require caution in their interpretation, particularly with respect to the sign of estimated biases. We found no relationship between emotion recognition biases and depression or anxiety scores using either morphed stimuli. We speculate that this may reflect elevated rates of depression and anxiety due to the COVID-19 pandemic and lockdowns, which may have masked such associations. Although emotion recognition bias tasks are useful tools in understanding the mechanisms underlying psychopathology, this study highlights the importance of considering methodology used and may explain some of the discrepancies in the literature.

## Supplementary Information

ESM 1 (DOCX 176 kb)

## Data Availability

The original materials are available at https://www.chicagofaces.org/ Morphed images are available at https://www.chicagofaces.org/resources/ The data are available at https://osf.io/9vw2g/?view_only=aaa543919f9c4975ba29153d781ce4f1
