# Supplementary material for: Emotion recognition bias depends on stimulus morphing strategy
Source: Atten Percept Psychophys. 2022 Jul 5;84(6):2051–9. doi: 10.3758/s13414-022-02532-0 (PMC9255837; doi:10.3758/s13414-022-02532-0)
Supplement: Supplementary file 1 — (DOCX 176 kb) [file 13414_2022_2532_MOESM1_ESM.docx]

**Table S1**

*Selected stimuli*

| Stimulus name | Stimulus description |
| --- | --- |
| BF002 | Black female |
| BF013 | Black female |
| BF018 | Black female |
| BF039 | Black female |
| BF040 | Black female |
| BM002 | Black male |
| BM026 | Black male |
| BM036 | Black male |
| BM040 | Black male |
| BM046 | Black male |
| WF003 | White female |
| WF006 | White female |
| WF020 | White female |
| WF022 | White female |
| WF039 | White female |
| WM024 | White male |
| WM025 | White male |
| WM033 | White male |
| WM034 | White male |
| WM038 | White male |

*Note.* All stimuli were selected from Chicago Face Database (CFD; Ma, Correll & Wittenbrink, 2015). Available upon request at: <https://www.chicagofaces.org/>

**Table S2**

*Correlations between emotion recognition bias for two morph types (angry-happy and angry-neutral-happy) and depression (PHQ-9) and anxiety (GAD-7).*

| Morph type | PHQ-9 | | | GAD-7 | | |
| --- | --- | --- | --- | --- | --- | --- |
|  | *r* | *p* | *95% CI* | *r* | *p* | *95% CI* |
| Angry-happy | .04 | .618 | [-0.15, 0.23] | -.02 | .793 | [-0.21, 0.16] |
| Angry-neutral-happy | -.06 | .492 | [-0.27, 0.14] | .02 | .865 | [-0.17, 0.20] |

*Note*. PHQ-9 = Patient Health Questionnaire, GAD-7 = Generalized Anxiety Disorder Scale.

* *p* < .025 (alpha corrected for two comparisons)

**Figure S1**

*Mean response times for each morphing level for morphing-through-neutral*

*
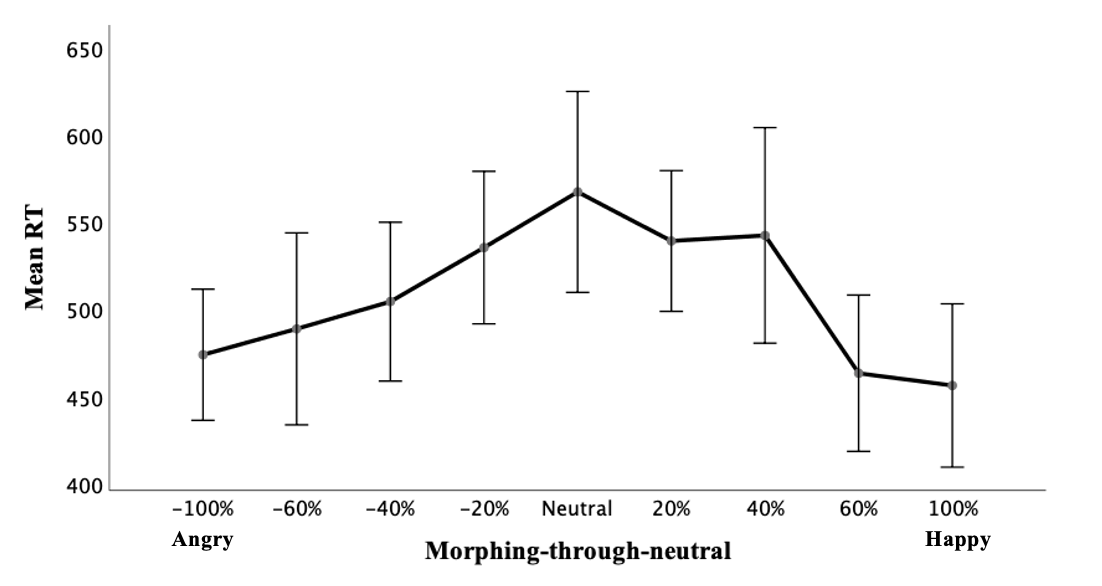
*

*Note.* Error bars indicate 95% confidence intervals.

**Figure S2**

*Mean response times for each morphing level for direct-morphing*

*
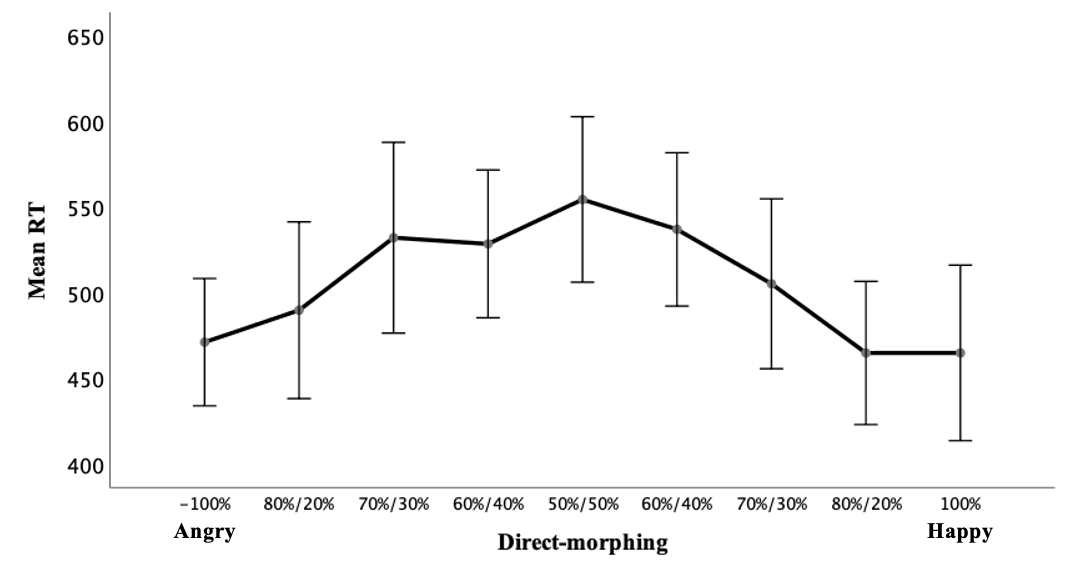
*

*Note.* Error bars indicate 95% confidence intervals.
